# Association between height and the risk of primary brain malignancy in adults: a nationwide population-based cohort study

**DOI:** 10.1093/noajnl/vdab098

**Published:** 2021-07-08

**Authors:** Stephen Ahn, Kyungdo Han, Jung Eun Lee, Sin-Soo Jeun, Yong Moon Park, Wonil Joo, Seung Ho Yang

**Affiliations:** 1 Department of Neurosurgery, Seoul St. Mary’s Hospital, College of Medicine, The Catholic University of Korea, Seoul, Korea; 2 Department of Statistics and Actuarial Science, Soongsil University, Seoul, Korea; 3 Department of Epidemiology, Branch, Fay W. Boozman College of Public Health, University of Arkansas for Medical Sciences, Little Rock, Arkansas, USA; 4 Department of Neurosurgery, Eunpyeong St. Mary’s Hospital, College of Medicine, The Catholic University of Korea, Seoul, Korea; 5 Department of Neurosurgery, St. Vincent’s Hospital, College of Medicine, The Catholic University of Korea, Seoul, Korea

**Keywords:** glioma, height, Koreans, primary brain malignancy, risk factors

## Abstract

**Background:**

The association between height and the risk of developing primary brain malignancy remains unclear. We evaluated the association between height and risk of primary brain malignancy based on a nationwide population-based database of Koreans.

**Methods:**

Using data from the Korean National Health Insurance System cohort, 6 833 744 people over 20 years of age that underwent regular national health examination were followed from January 2009 until the end of 2017. We documented 4771 cases of primary brain malignancy based on an ICD-10 code of C71 during the median follow-up period of 7.30 years and 49 877 983 person-years.

**Results:**

When dividing the population into quartiles of height for each age group and sex, people within the highest height quartile had a significantly higher risk of brain malignancy, compared to those within the lowest height quartile (HR 1.21 CI 1.18–1.32) after adjusting for potential confounders. We also found that the risk of primary brain malignancy increased in proportion with the quartile increase in height. After analyzing subgroups based on older age (≥ 65) and sex, we found positive relationships between height and primary brain malignancy in all subgroups.

**Conclusions:**

This study is the first to suggest that height is associated with an increased risk of primary brain malignancy in the East-Asian population. Further prospective and larger studies with precise designs are needed to validate our findings.

**Key Points**

- The associations between height and common cancer types have been well established.
- However, the association between height and primary brain malignancy remains unclear.
- This study showed that taller people had a higher risk of primary brain malignancy.

**Importance of the Study**

While height is a well-known risk factor for developing various types of cancers, conflicting results have been reported concerning the relationship between height and the risk of primary brain malignancy. To date, no studies have evaluated this association in Eastern-Asian populations, who are unique and known to usually be shorter than Western populations, although the average height of Koreans has rapidly increased over the last century. This nationwide population-based study is the first study to show the positive association of height with the risk of primary brain malignancy in the Eastern-Asian population. This study has also adjusted numerous possible confounders such as smoking and drinking habits, socioeconomic status, and underlying diseases to provide consolidative results for identifying the risk of height for developing primary brain malignancy.

Primary brain malignancy is the second most common primary brain tumor in adults.^1^ Adult glioma represents more than 90% of all brain malignancies, which are mainly composed of glioblastomas, diffuse astrocytomas, oligodendrogliomas, and ependymomas.^2^ The prognoses are usually fatal, and for cases of glioblastoma, which is the most common malignancy among all gliomas, most patients eventually die within 5 years of diagnosis.^3^ Over the last decade, molecular features of tumorigenesis in primary malignant brain tumors have been largely explored^4^; however, risk factors such as anthropometric measurements, lifestyle habitus, and environmental exposure, have been poorly defined.^5^

Over the last decade, height, which is an easily measured anthropometric measurement, has been firmly established as a significant risk factor for various cancer types in addition to obesity.^6,7^ Biological findings that increased insulin-like growth factors (IGF) were measured in taller population groups and it was determined that IGF could facilitate tumor cell growth, which has supported this epidemiological association.^8^

However, conflicting results have been observed regarding the risk of developing primary brain malignancy.^9–17^ Several previous studies showed that height was significantly associated with glioma development^9,11,13–15^; however, a few studies, including a recent large study reported a null association.^10,12,16,17^ To the best of our knowledge, all of these studies included only Western populations. Eastern-Asian populations are unique and known to usually be shorter than Western populations, although the average height of Koreans has rapidly increased over the last century.^18,19^

In this context, we evaluated the potential associations between height and the risk of primary brain malignancy in adults, using a nationwide population database obtained from the National Health Insurance Service (NHIS) of Koreans. This nationwide database included detailed information for numerous possible confounders such as smoking status, socioeconomic status, and underlying disease, which have already provided powerful and consolidated results for identifying risk factors in various cancer types.^6,20^

## Materials and Methods

### Ethical Statement

The Institutional Review Board of Seoul St. Mary’s Hospital approved this study design (ethical code: KC18ZESI0648, permission date: 23 October 2018). This study was conducted in accordance with the ethical standards of the 1964 Declaration of Helsinki. To protect individuals’ information, all data were anonymized. Due to the retrospective manner of this study, the requirement for informed consent was waived.

### Database Source

This retrospective nationwide population-based cohort study was performed using the NHIS of Korea as the source database, which is a mandatory health insurance system operated by the Korean government and that covers almost all Koreans, accounting for approximately 50 million people. The NHIS database included demographic information such as age, sex, and income level. Medical information such as clinical diagnoses, prescribed medication, and surgical procedures are included. In addition, information obtained from regular national health examination, which is regularly provided by the NHIS either for all enrolled adults > 40 years old at least every two years, or for any workers at a company > 20 years old was included. During national health examination, physical measurements including height and weight were collected and blood samples including complete blood counts, glucose level, and lipid profiles, and blood pressure were measured by the healthcare providers. Using self-reported questionnaire records, socio-behavioral history including cigarette smoking, alcohol consumption, and physical activity, and family history were also obtained.

### Study Population

**Figure 1.**
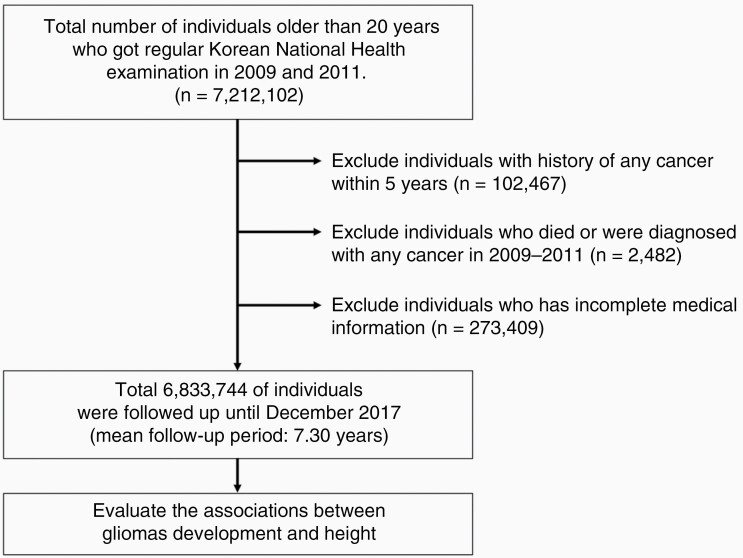
Flow of study design.

We reviewed records from the NHIS database for all people that were over 20 years of age as of 2009. We were able to access the database of a total of 10 million people due to the personal information protection policy of the NHIS. Among these individuals, we included individuals who underwent a regular national health examination annually or biennially both in 2009 and 2011 and excluded individuals with any history of cancer within 5 years, or that had a documented death for any reason, or that were diagnosed with any cancer in the 2-year lag period, and/or with incomplete medical information. After enrolling the study population based on our inclusion and exclusion criteria, 6 833 744 people were identified. We followed up this population from January 2009 to December 2017. The mean follow-up periods for enrolled individuals were 7.30 years and 49 877 983 person-years. The flow of the study population was described in Figure 1.

### Definition of Primary Brain Malignancy

We already described the definition of primary brain malignancy in our previous publication.^20^ Briefly, the medical code “C71” represents malignant neoplasms of the brain, according to the International Classification of Disease, Tenth Revision (ICD-10), and all C71 patients of Korean received an additional cost coverage service from the NHIS for rare and incurable diseases. Using this, we defined patients with primary brain malignancy as those who were diagnosed both with C71 and who were registered with a benefits extension policy for rare incurable diseases. To verify the accuracy of our method of identification, we retrospectively reviewed the electronic medical records at Seoul St. Mary’s Hospital, a tertiary referral hospital in Korea. After evaluating medical records for patients older than 18 years who fit our definition of a confirmed primary brain malignancy between 2014 and 2018, we confirmed that 100% of these patients had been diagnosed with glioma either pathologically or radiologically (Supplementary Table 1).

### Clinical Variables

Because height is dependent on age and sex, we divided the population into quartiles of height for each age group (20–29 years, 30–39 years, 40–49 years, 50–59 years, 60–69 years, and ≥70 years) and sex (Supplementary Table 2). We analyzed these merged quartile groups for incidence and hazard ratios for glioma. In addition, the definition of clinical variables including smoking status, alcohol status, exercise, and underlying diseases was described in our previous studies.^20,21^

### Statistical Analyses

Data are expressed as mean ± standard deviation for continuous variables and as proportions for categorical variables. One-way analysis of variance (ANOVA) was used to compare differences between continuous variables, and the chi-square test was used to compare differences between categorical variables. Incidence rates for primary brain malignancies were calculated and expressed as the number of events per 100 000 person-years. Cumulative incidence rates for primary brain malignancy were compared between groups using the Kaplan–Meier method and the log-rank test. With the diagnosis of primary brain malignancy or death was founded, the censoring was done. Cox proportional hazards models were used to analyze the adjusted risk of primary brain malignancy, based on height; results are expressed as hazard ratios (HRs) with 95% confidence intervals (CIs). We fit a model adjusted for the potential confounders of age, sex, smoking status, alcohol consumption, exercise level, body mass index, and presence of diabetes mellitus (adjusted model). A *P*-value <.05 was considered statistically significant. Statistical analyses were performed using SAS version 9.4 (SAS Institute, Cary, NC).

## Results

### Study Population

A total of 4471 cases of primary brain malignancy among 6,833,744 individuals developed during the follow-up period of 49 877 983 person-years. The incidence rate of primary brain malignancy was 8.96 per 100 000 person-years in our study population. Baseline characteristics including age, sex, height, body mass index (BMI), smoking status, alcohol consumption, physical activity, diabetes mellitus, hypertension, dyslipidemia, and income level were collected. This information was described according to age- and sex-adjusted quartiles of height in Table 1.

**Table 1.** Baseline Characteristics of the Study Population

*n* (%)	Quartile 1	Quartile 2	Quartile 3	Quartile 4	*P* Value
	*n* = 1 751 006	*n* = 1 694 470	*n* = 1 696 440	*n* = 1 691 828	
Mean age, years^a^	47.0 ± 13.4	47.5 ± 13.8	46.56 ± 13.16	46.2 ± 13.7	<.001
Male	1 013 864 (57.9)	989 953 (58.42)	925 692 (54.57)	996 110 (58.88)	<.001
BMI, kg/m^2^^a^	23.9 ± 4.3	23.8 ± 3.1	23.7 ± 3.14	23.7 ± 3.2	<.001
<18.5	50 097 (2.86)	53 566 (3.16)	56 552 (3.33)	63 670 (3.76)	
18.5–22.9	659 985 (37.69)	639 943 (37.77)	666 125 (39.27)	658 066 (38.9)	
23.0–24.9	451 163 (25.77)	431 286 (25.45)	424 437 (25.02)	423 796 (25.05)	
25.0–29.9	528 391 (30.18)	514 181 (30.34)	492 465 (29.03)	488 663 (28.88)	
30.0 ≤	61 370 (3.5)	55 494 (3.28)	56 861 (3.35)	57 633 (3.41)	
Smoker					<.001
None	1 049 061 (59.91)	980 186 (57.85)	1 006 268 (59.32)	945 350 (55.88)	
Former	244 908 (13.99)	269 085 (15.88)	256 870 (15.14)	283 533 (16.76)	
Current	457 037 (26.1)	445 199 (26.27)	433 302 (25.54)	462 945 (27.36)	
Drinker					<.001
None	905 037 (51.69)	846 532 (49.96)	855 241 (50.41)	808 299 (47.78)	
Mild	718 970 (41.06)	714 148 (42.15)	709 977 (41.85)	733 405 (43.35)	
Heavy	126 999 (7.25)	133 790 (7.9)	131 222 (7.74)	150 124 (8.87)	
Regular exercise	299 601 (17.11)	318 857 (18.82)	323 608 (19.08)	341 304 (20.17)	<.001
Diabetes mellitus	143 735 (8.21)	146 035 (8.62)	131 409 (7.75)	138 096 (8.16)	<.001
Fasting Glucose	96.74 ± 22.96	97.1 ± 22.9	96.6 ± 22.1	97.0 ± 22.5	
Hypertension	253 511 (14.48)	246 326 (14.54)	228 490 (13.47)	231 667 (13.69)	<.001
Systolic BP^a^	122.44 ± 15.05	122.6 ±14.9	122.1 ± 14.6	122.5 ± 14.4	
Diastolic BP^a^	76.4 ± 10	76.5 ± 9.9	76.2 ± 9.9	76.5 ± 9.8	
Dyslipidemia	327 780 (18.72)	318 942 (18.82)	300 307 (17.7)	287 938 (17.02)	< 0.001
Total cholesterol	197.09 ± 41.38	196.2 ± 40.6	195.5 ± 40.4	193.8 ± 40.5	
HDL	56.62 ± 32.93	56.3 ± 32.5	56.4 ± 33.7	55.9 ± 31.7	
LDL	123.5 ± 226	124.0 ± 242.3	122.8 ± 228.6	122.9 ± 249.9	
Low-income level	399 500 (22.82)	347 453 (20.51)	333 427(19.65)	312 813 (18.49)	<.001

BMI, body mass index; BP, blood pressure; HDL, high-density lipoprotein; LDL, low-density lipoprotein.

^a^Described as mean ± standard deviation.

### Risks of Adult Glioma According to Quartile of Height

Individuals within the highest (Q4) and second highest (Q3) quartiles of height had a significantly higher risk of primary brain malignancy, compared to individuals within the lowest quartile of height (HR 1.21 CI 1.18–1.32 for Q4 and HR 1.13 CI 1.04–1.23 for Q3) after adjusting for age, sex, body mass index, smoking status, alcohol consumption, physical activity, the presence of diabetes mellitus, and income level (Table 2). The Kaplan-Meier curve for a cumulative incidence of primary brain malignancy according to quartile of height was illustrated in Supplementary Figure 1. In addition, height per cm increase was significantly associated with primary brain malignancy (HR 1.01, CI 1.01–1.02).

**Table 2.** Risk of Primary Brain Malignancy According to Quartile of Height

Height	Total, *n*	Events, *n*	Person-years	Incidence Rate^a^	Unadjusted HR (95% CI)	Adjusted HR^b^ (95% CI)
Quartile 1	1 751 006	1041	12 766 913	8.15	1 (Reference)	1 (Reference)
Quartile 2	1 694 470	1137	12 366 463	9.19	1.13 (1.04–1.23)	1.08 (1.00–1.18)
Quartile 3	1 696 440	1105	12 389 684	8.92	1.01 (1.01–1.19)	1.13 (1.04–1.23)
Quartile 4	1 691 828	1188	12 354 925	9.62	1.18 (1.09–1.28)	1.21 (1.18–1.32)

CI, confidence interval; HR, hazard ratio.

^a^Per 100 000 person-years.

^b^Adjusted for sex, age, body mass index, smoking status, alcohol consumption, exercise level, income level, and the presence of diabetes mellitus.

### Subgroup Analyses According to Age and Sex

After analyzing subgroups according to older age (≥ 65 years) and sex, we also identified the positive relationships between height and primary brain malignancy in all subgroups (Table 3). The highest (Q4) and second highest (Q3) quartile of height in other subgroups were also at significantly higher risk for developing primary brain malignancy, whereas only the highest (Q4) quartile of height in female subgroups was at a significantly higher risk of primary brain malignancy.

**Table 3.** Subgroup Analyses of Risk of Primary Brain Malignancy, According to Age and Sex Group

	Height	Total, *n*	Events, *n*	Person- years	Incidence Rate^a^	Unadjusted HR (95% CI)	Adjusted HR^b^ (95% CI)
Age < 65							
	Quartile 1	1 546 938	724	11 334 204	6.39	1 (Reference)	1 (Reference)
	Quartile 2	1 475 944	746	10 818 625	6.90	1.08 (0.98–1.20)	1.05 (0.95–1.17)
	Quartile 3	1 511 233	753	11 079 774	6.80	1.06 (0.96–1.18)	1.11 (1.00–1.23)
	Quartile 4	1 511 503	854	11 077 724	7.71	1.21 (1.09–1.33)	1.22 (1.11–1.35)
Age ≥ 65	Quartile 1	204 068	317	1 432 709	22.13	1 (Reference)	1 (Reference)
	Quartile 2	218 526	391	1 547 838	25.26	1.14 (0.98-0.32)	1.16 (1.00–1.34)
	Quartile 3	185 207	352	1 309 908	26.87	1.21 (1.04–1.41)	1.21 (1.04–1.41)
	Quartile 4	180 325	334	1 277 200	26.15	1.18 (1.01–1.38)	1.19 (1.07–1.38)
Male							
	Quartile 1	1 013 864	591	7 359 831	8.03	1 (Reference)	1 (Reference)
	Quartile 2	989 953	659	7 196 010	9.16	1.14 (1.02–1.27)	1.10 (0.99–1.23)
	Quartile 3	925 692	619	6 734 174	9.19	1.15 (1.02–1.28)	1.19 (1.07–1.34)
	Quartile 4	996 110	684	7 251 240	9.43	1.18 (1.05–1.31)	1.21 (1.09–1.35)
Female							
	Quartile 1	737 142	450	5 407 082	8.32	1 (Reference)	1 (Reference)
	Quartile 2	704 517	478	5 170 453	9.25	1.11 (0.98–1.26)	1.07 (0.94–1.21)
	Quartile 3	770 748	486	5 655 509	8.59	1.03 (0.91–1.17)	1.06 (0.93–1.21)
	Quartile 4	695 718	504	5 103 684	9.87	1.19 (1.05–1.35)	1.22 (1.08–1.39)

CI, confidence interval; HR, hazard ratio.

^a^Per 100 000 person-years.

^b^Adjusted for sex, age, body mass index, smoking status, alcohol consumption, exercise level, income level, and the presence of diabetes mellitus.

## Discussion

In this nationwide population-based cohort study, we showed that taller individuals were at significantly higher risk for developing primary brain malignancy. When the population was divided into quartiles of height for each age group and sex, individuals within the highest quartile of height had a significantly higher risk of brain malignancy, compared to individuals within the lowest quartile of height (HR 1.21 CI 1.18–1.32). We showed that the risk of brain malignancy increased as each quartile of height increased (HR 1.21 CI 1.18–1.32 for Q4 and HR 1.13 CI 1.04–1.23 for Q3). Because the average height can differ among individuals depending on sex and age groups, we also evaluated these associations in subgroup analyses of sex and age. Similarly, we confirmed the positive relationships between height and primary brain malignancy in younger groups (< 65), elderly groups (≥ 65), male groups, and female groups. To the best of our knowledge, this is the first study to show that taller individuals in the East-Asian population had a significantly higher risk of developing primary brain malignancy as adults.

Biological studies have suggested potential mechanisms to support these associations.^8,22–24^ IGF, which is produced by growth hormone stimulation in the liver, is known to impact cell proliferation of both normal cells and cancer cells.^8^ Previous studies showed that significantly increased levels of IGF are observed in taller individuals, compared with shorter individuals.^23,24^ These increased circulating levels of IGF observed in taller individuals during childhood and adolescence have been suggested as a potential pathophysiological mechanism for the link between height and the risk of common cancer types.^22–24^ In addition, insulin-like growth factor binding protein-2 (IGFBP-2), which is expressed higher in taller people, as is IGF, is suggested as a biomarker to connect height and glioma development.^21,25^ As an engagement molecule to bind IGF, overexpression of IGFBP-2, which is found in most glioblastoma, is considered a biomarker representing aggressiveness and is suggested as a key signal of gliomagenesis.^26,27^

Epidemiolocal studies have provided strong evidence to show positive associations between height and the risk of cancers,^6,7,28^ however, the results remain controversial for primary brain malignancy.^9–17^ While previous studies showed positive associations between height and glioma development,^9,11,13–15^ a few studies, including a recent and large study that used Mendelian analysis, showed null associations.^10,12,16,17^ The rare incidence of primary brain malignancy is one possible reason for these conflicting results. In addition, the potential risk factors for developing gliomas are poorly defined, and most studies were not able to account for these various potential risk factors.^5,17^ Compared with previous studies, our nationwide cohort study included a larger number of primary brain malignancy cases (*n* = 4,471), whereas most studies to date have included less than 2000 cases.^9–11,13,16^ Moreover, this nationwide database operated by the NHIS of the Korean government provided detailed information, especially about possible confounders including obesity, alcohol habitus, smoking status, social-economic status, and the presence of metabolic diseases.^20,29^ Hazard ratios of the possible confounders included in our adjusted model were described in Supplementary Table 3. Using information from this database has allowed powerful and consolidative results for identifying risk factors in various cancer types, which have already been published.^6,20,29^

Our findings should be considered within the scope of several limitations. “First, primary brain malignancy in adults is comprised of heterogeneous subtypes, but analysis according to subtype was not possible in our study. Prior studies suggested that height as a glioma risk factor was stronger in glioblastoma cases, while this association was not significant in restricting isocitrate dehydrogenase (IDH) mutant gliomas.^11,15^ Further studies are needed to analyze the incidence and risk factors according to histologic and molecular subgroups.” Second, although previous studies showed diabetes might be unrelated to glioma, our study results did not agree.^30^ To reduce bias, our adjusted model included the presence of diabetes. Third, we verified the definition of primary brain malignancy by retrospectively reviewing electronic medical records at a tertiary referral hospital in Korea, and all tumors that were identified aligned with our defined method, which was all gliomas. However, these results from a tertiary hospital may not be generalizable to other hospitals in Korea. Lastly, although relatively few individuals (*n* = 273 409) were excluded due to incomplete medical information compared with a whole population (*n* = 7 212 102), this missing data might have influenced our results.

In conclusion, further prospective and larger epidemiological studies are to validate our findings. Especially, further studies that include histological and molecular subgroups are recommended. In addition to epidemiological studies, biological studies to elucidate potential mechanisms between increased IGF and gliomagenesis are also needed.

## Supplementary Material

vdab098_suppl_Supplementary_Figure_S1

vdab098_suppl_Supplementary_Materials

## Data Availability

Data available on request due to privacy/ethical restrictions.
