# Supplementary figures and images for: Association between height and the risk of primary brain malignancy in adults: a nationwide population-based cohort study
Source: Neurooncol Adv. 2021 Jul 8;3(1):vdab098. doi: 10.1093/noajnl/vdab098 (PMC8562729; doi:10.1093/noajnl/vdab098)

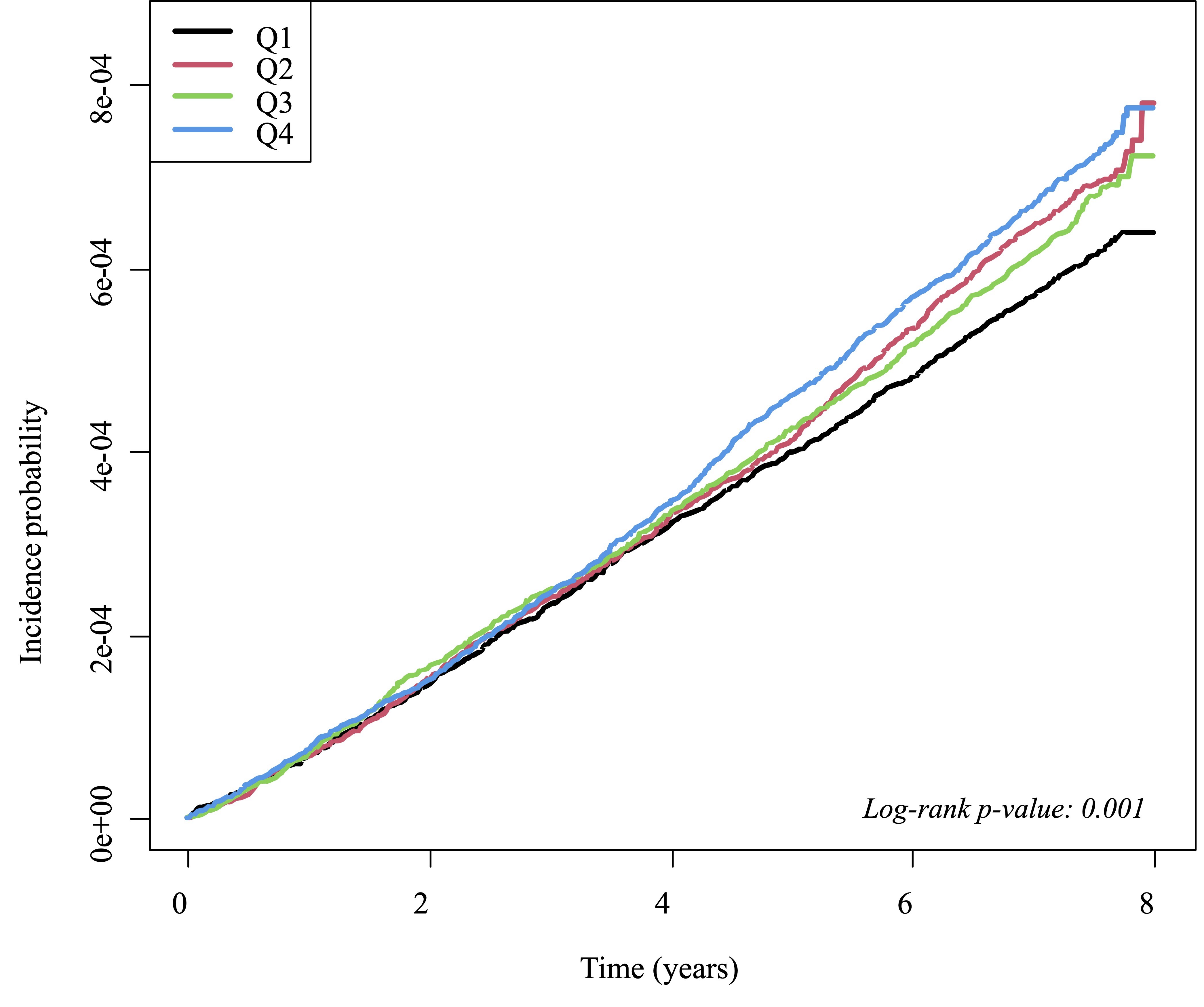

Supplement: vdab098_suppl_Supplementary_Figure_S1 [file vdab098_suppl_supplementary_figure_s1.jpeg]
