# Supplementary material for: Association between height and the risk of primary brain malignancy in adults: a nationwide population-based cohort study
Source: Neurooncol Adv. 2021 Jul 8;3(1):vdab098. doi: 10.1093/noajnl/vdab098 (PMC8562729; doi:10.1093/noajnl/vdab098)
Supplement: vdab098_suppl_Supplementary_Materials [file vdab098_suppl_supplementary_materials.docx]

**Supplementary table 1.** Characterization of adult patients with C71 code in Seoul St. Mary’s Hospital between 2014 and 2018.

|  | Total = 220, n (%) |
| --- | --- |
| Male | 117 (53.2%) |
| Mean age, years (range) | 51.3 (20-80) |
| Diagnosis |  |
| Anaplastic astrocytoma (Grade III) | 18 (8.2) |
| Anaplastic ependymoma (Grade III) | 9 (4.1) |
| Anaplastic oligodendroglioma (Grade III) | 15 (6.8) |
| Anaplastic oligoastrocytoma (Grade III) | 6 (2.7) |
| Diffuse astrocytoma (Grade II) | 10 (4.5) |
| Ependymoma (Grade II) | 7 (3.2) |
| Glioblastoma (Grade IV) | 113 (51.4) |
| Oligodendroglioma (Grade II) | 22 (10.0) |
| Oligoastrocytoma (Grade II) | 3 (1.4) |
| Pleomorphic xanthoastrocytoma (Grade II) | 3 (1.4) |
| Low grade glioma* | 9 (4.1) |
| High grade glioma* | 5 (2.3) |

*Radiologically diagnosed only.

**Supplementary table 2.** Reference value of height quartile based on age and sex groups.

| Sex | Age Group | Height (cm) | | | |
| --- | --- | --- | --- | --- | --- |
|  | (years) | Quartile 1 | Quartile 2 | Quartile 3 | Quartile 4 |
| Male | 20-29 | < 170 | 170-174 | 174-177 | 178 ≤ |
|  | 30–39 | < 169 | 169-172 | 172-176 | 176 ≤ |
|  | 40-49 | < 166 | 166-170 | 170-174 | 175 ≤ |
|  | 50-59 | < 164 | 164-168 | 171-172 | 172 ≤ |
|  | 60-69 | < 162 | 162-166 | 169-170 | 170 ≤ |
|  | 70-79 | < 160 | 160-164 | 168-169 | 169 ≤ |
|  | 80 ≤ | < 158 | 158-162 | 162-166 | 167 ≤ |
| Female | 20-29 | < 157 | 157-161 | 161-164 | 165 ≤ |
|  | 30–39 | < 156 | 156-159 | 159-163 | 164 ≤ |
|  | 40-49 | < 154 | 154-157 | 157-161 | 162 ≤ |
|  | 50-59 | < 152 | 152-155 | 159-160 | 160 ≤ |
|  | 60-69 | < 149 | 149-153 | 153-156 | 157 ≤ |
|  | 70-79 | < 146 | 146-150 | 154-155 | 155 ≤ |
|  | 80 ≤ | < 143 | 143-147 | 147-151 | 152 ≤ |

**Supplementary Table 1.** Risk of primary brain malignancy according to possible confounders

| **Variables** | **Total, n** | **Events, n** | **Person-years** | **Incidence rate*** | **Adjusted HR^†^ (95% CI)** |
| --- | --- | --- | --- | --- | --- |
| Age per a year |  |  |  |  | 1.06 (1.05-1.06) |
| Sex |  |  |  |  |  |
| Male | 3925619 | 2553 | 28541255 | 8.94 | 1 (Reference) |
| Female | 2908125 | 1,918 | 21336727 | 8.99 | 0.84 (0.78-0.91) |
| Body mass index per kg/m^2^ |  |  |  |  | 1.00 (1.00-1.004) |
| Income |  |  |  |  |  |
| High | 5440551 | 3543 | 39709820 | 8.92 | 1 (Reference) |
| Low | 1393193 | 928 | 10168162 | 9.13 | 1.00 (0.93-1.08) |
| Smoking status |  |  |  |  |  |
| None | 3980865 | 2659 | 29138206 | 9.13 | 1 (Reference) |
| Former | 1054396 | 731 | 7675094 | 9.52 | 1.02 (0.93-1.13) |
| Current | 1798483 | 1081 | 13064681 | 8.27 | 1.25 (1.15-1.37) |
| Drinker |  |  |  |  |  |
| None | 3415109 | 2631 | 24923832 | 10.56 | 1 (Reference) |
| Mild | 2876500 | 1502 | 21017024 | 7.15 | 0.90 (0.84-0.97) |
| Heavy | 542135 | 338 | 3937125 | 8.59 | 0.95 (0.84-1.07) |
| Regular exercise |  |  |  |  |  |
| No | 5550374 | 3576 | 40490331 | 8.83 | 1 (Reference) |
| Yes | 1283370 | 895 | 9387651 | 9.53 | 0.96 (0.89-1.04) |
| Diabetes mellitus |  |  |  |  |  |
| No | 6274469 | 3860 | 45870376 | 8.42 | 1 (Reference) |
| Yes | 559275 | 611 | 4007607 | 15.25 | 1.11 (1.01-1.21) |

CI, confidence interval; HR, hazard ratio; n, number

*per 100,000 person-years

^†^adjusted for sex, age, body mass index, smoking status, alcohol consumption, exercise level, income level, and the presence of diabetes mellitus.
